# The relationship between high ratios of CD4/FOXP3 and CD8/CD163 and the improved survivability of metastatic triple-negative breast cancer patients: a multicenter cohort study

**DOI:** 10.1186/s13104-024-06704-z

**Published:** 2024-02-02

**Authors:** Jeffry Beta Tenggara, Andhika Rachman, Joedo Prihartono, Lisnawati Rachmadi, Sonar Soni Panigoro, Didik Setyo Heriyanto, Noorwati Sutandyo, Intan Russianna Nasution, Familia Bella Rahadiati, Ricci Steven, Rachelle Betsy, Samuel Juanputra, Aru Wisaksono Sudoyo

**Affiliations:** 1 Division of Hematology and Medical Oncology, Department of Internal Medicine, Dr. Cipto Mangunkusumo General Hospital–Faculty of Medicine Universitas Indonesia, Jl. Pangeran Diponegoro No. 71, RW.5, Kec. Senen, Central Jakarta, Jakarta, 10430 Indonesia; 2 Division of Hematology and Medical Oncology, Department of Internal Medicine, MRCCC Siloam Hospital Jakarta, Jakarta, Indonesia; 3 Department of Community Medicine, Dr. Cipto Mangunkusumo General Hospital–Faculty of Medicine, Universitas Indonesia, Jakarta, Indonesia; 4 Department of Anatomical Pathology, Dr. Cipto Mangunkusumo General Hospital–Faculty of Medicine, Universitas Indonesia, Jakarta, Indonesia; 5 Department of Surgical Oncology, Dr. Cipto Mangunkusumo General Hospital–Faculty of Medicine, Universitas Indonesia, Jakarta, Indonesia; 6 Department of Anatomical Pathology, Dr. Sardjito Hospital–Faculty of Medicine, Public Health and Nursing, Universitas Gadjah Mada, Yogyakarta, Indonesia; 7 Division of Hematology and Medical Oncology, Department of Internal Medicine, Dharmais National Cancer Hospital, Jakarta, Indonesia; 8 Division of Hematology and Medical Oncology, Gatot Soebroto Army Hospital Jakarta, Jakarta, Indonesia; 9 Department of Anatomical Pathology, Gatot Soebroto Army Hospital Jakarta, Jakarta, Indonesia; 10 Department of Internal Medicine, Dr. Cipto Mangunkusumo General Hospital–Faculty of Medicine, Universitas Indonesia, Jakarta, Indonesia

**Keywords:** Survival, Metastatic, Triple-negative breast cancer, CD4, CD8, CD163, FOXP3

## Abstract

**Background:**

Triple-negative breast cancer (TNBC) has been documented as the most aggressive subtype of breast cancer. This study aimed to analyze antitumor and protumor immune activities, and their ratios as significant prognostic biomarkers in metastatic TNBC (mTNBC).

**Methods:**

A multicenter cohort study was conducted among 103 de novo mTNBC patients. The expression of CD8 and CD163 was evaluated using immunohistochemistry staining, CD4 and FOXP3 using double-staining immunohistochemistry, and PD-L1 using immunohistochemistry and RT-PCR.

**Results:**

Multivariate analysis revealed that high CD4/FOXP3 (HR 1.857; 95% CI 1.049–3.288; *p* = 0.034) and the CD8/CD163 ratio (HR 2.089; 95% CI 1.174–3.717; *p* = 0.012) yield significantly improved 1 year overall survival (OS). Kaplan–Meier analysis showed that high levels of CD4 (*p* = 0.023), CD8 (*p* = 0.043), CD4/FOXP3 (*p* = 0.016), CD8/FOXP3 (*p* = 0.005), CD8/CD163 (*p* = 0.005) ratios were significantly associated with higher rate of 1 year OS. Furthermore, 1 year OS was directly correlated with antitumor CD4 (R = 0.233; *p* = 0.018) and CD8 (R = 0.219; *p* = 0.026) and was indirectly correlated with protumor CD163 and FOXP3 through CD4/FOXP3 (R = 0.282; *p* = 0.006), CD4/CD163 (R = 0.239; *p* = 0.015), CD8/FOXP3 (R = 0.260; *p* = 0.008), and CD8/CD163 (R = 0.258; *p* = 0.009).

**Conclusion:**

This is the first study to demonstrate that high levels of CD4/FOXP3 and CD8/CD163 significantly improved the 1 year OS in de novo mTNBC patients. Thus, we recommend the application of these markers as prognosis determination and individual treatment decision.

**Supplementary Information:**

The online version contains supplementary material available at 10.1186/s13104-024-06704-z.

## Introduction

Triple-negative breast cancer (TNBC) has been established as the most aggressive subtype of breast cancers and is frequently associated with inflammation in the stroma along with a greater risk of immune cell infiltration compared to other subtypes [1, 2]. The identification of TNBC is based on the cells expression of estrogen receptors (ER) and progesterone receptors (PR) of ≤ 1% and negative expression of human epidermal growth factor receptor 2 (HER2) [1]. The lack of ER, PR, and HER2 protein expression makes TNBC unresponsive to the current endocrine and HER2-targeted therapies and leads to a poor prognosis [1, 3]. TNBC is responsible for 12–17% of all breast cancers and is inevitably recurrent [1, 4]. Its incidence has been documented to be increasing consistently over the past few decades. The median survival for metastatic TNBC (mTNBC) is 13.3 months and the mortality rate at 12 months is 75% [1, 5–7].

Based on current evidence, tumor microenvironment (TME) plays a critical role in TNBC immunomodulation, which can be categorized as immunoreactive (antitumor) or immunosuppressive (protumor). Immunosuppressive TME is majorly comprised of forkhead-box-P3 (FOXP3) regulatory T-cells (Treg), M2 macrophages, and programmed death*-*ligand 1 (PD*-*L1) axis. Immunoreactive TME is mainly comprised of CD4 and CD8 T-cells, M1 macrophages, and natural killer (NK) cells [3].

We hypothesized that alterations in protumor and antitumor immune activities might impact disease progression in mTNBC [8]. We aimed to analyze the use of antitumor and protumor immune activities, and their ratios as prognostic markers in de novo mTNBC patients [9–11]. Thus, a comprehensive understanding of protumor and antitumor immune activities can be obtained as a promising strategy for evaluating the prognosis and treatment for mTNBC [9, 12].

## Methods

### Patient samples and methodology

A multicenter cohort study was conducted at two Indonesian National Cancer Centers (Dharmais National Cancer Hospital and Mochtar Riady Comprehensive Cancer Center), Cipto Mangunkusumo National Central General Hospital, Gatot Soebroto Central Army Hospital, Siloam Lippo Village Hospitals, Jakarta Breast Cancer Hospital, and Metropolitan Medical Center Hospital. The study population consisted of all patients diagnosed with mTNBC (stage IV) defined by immunohistochemistry (IHC) from available formalin-fixed paraffin-embedded (FFPE) tissue blocks from January 2015 to December 2020 [13, 14]. The included subjects ≥ 18 years old and their de novo mTNBC status was confirmed by histopathology and IHC with ER and PR < 1% and their HER2 receptors were at either 0, 1 + , or 2 + with non-amplified fluorescence in situ hybridization (FISH) test result, all of which were in accordance with American Society of Clinical Oncology (ASCO) guidelines [1, 3]. Exclusion criteria were either incomplete medical record data and/or unsuitable FFPE tissue samples for further examination.

### Analysis of tumor-infiltrating lymphocytes (TILs)

The infiltration of immune cells was analyzed on H and E stained slides [15, 16]. The average of total cell count from five fields with the highest concentration of TILs was quantified under 200 ×magnification [17]. All slides were examined by two pathologists who had extensive expertise in mammary pathology (L. and D.S.H.) [15, 18].

### IHC evaluation

The levels of CD4, CD8, FOXP3, and CD163 were evaluated in the immune cells located in the invasive tumor area [15]. CD8 and CD163 were demonstrated by staining methods using antibodies to CD8 (Cell Marque, 108R-14) and CD163 (Biocare Medical, ACR353AK) cells. FOXP3 and CD4 cells were evaluated by the double-staining method using antibodies to CD4 (Biocare Medical, ACI3148) and FOXP3 (Genetex, GTX107737) cells. The MACH 2 Double Stain 2 (Biocare Medical) was utilized for the incubation process. FOXP3 and CD4 cells were stained with Vulcan Fast Red (Biocare Medical) [17].

Immunohistochemical staining of PD-L1 was performed using a mouse monoclonal primary anti-PD-L1 antibody (clone 22C3; Dako; Agilent Technologies, Inc.). Subsequently, the slides were incubated with Novolink Polymer Detection System (Leica Microsystems) as a secondary antibody (Novocastra). The combined positive score (CPS) was used for evaluating immunohistochemical expression of PD-L1 [19].

### PD-L1 mRNA

The mRNA samples were analyzed using the NEXproTM qRT-PCR Master Mix (SYBR) kit in accordance with the manufacturer’s instructions. The Bioneer Exicycler^™^ 96 Real-Time Quantitative Thermal Block was used for the quantitative PCR which was performed in accordance with the manufacturer’s instructions [20]. PCR primers were as follows: forward 5′—TATGGTGGTGCCGACTACAA-3′ and reverse 5′—TGGCTCCCAGAATTACCAAG-3' [21].

### Statistical analysis

The extracted data was analyzed with Statistical Package for the Social Sciences (SPSS) version 27 for Windows. ROC curve was used to determine the optimal cut-off for categorizing the low and high levels groups. Survival analysis were performed using Kaplan–Meier and Cox proportional hazard models. Spearman correlation was used to analyze the correlation between each prognostic marker and the 1 year OS.

## Results

Initially, 128 female subjects with de novo mTNBC who fulfilled all inclusion and exclusion criteria were recruited. Among them, 103 subjects were included for IHC and RT-PCR evaluation (Additional file 1: Fig. S1).

The mean age was 51.3 years old, while the mean body mass index (BMI) was 23.2. The most frequent sites of metastasis are the lung (56.3%) and bone (49.5%). Histopathology characteristics showed most subjects had NST type (92.2%), grade III (54.4%), and high Ki-67 (86.4%). The chemotherapy agents were used according to National Comprehensive Cancer Network in oncology (NCCN) guidelines Table 1, [22]. The IHC staining and double-staining are depicted in (Additional file 2: Fig. S2).

**Table 1:** Subject characteristics

Characteristics	N (103)
Age, mean ± SD, in years	51.3 ± 12.6
BMI, mean ± SD, in kg/m^2^	23.2 ± 6.1
Chemotherapy, N (%)	
Antimetabolite (5-FU, capecitabine, gemcitabine, methotrexate)	41 (39.8)
Anthracycline (doxorubicin, epirubicin)	40 (38.8)
Alkylating agents (cyclophosphamide)	34 (33)
Taxane (docetaxel, paclitaxel)	36 (34.9)
Platinum (carboplatin, cisplatin)	23 (22.3)
Vinca alkaloid (vinorelbine)	2 (1.9)
Antimicrotubule (eribulin)	2 (1.9)
Histopathology, N (%)	
NST	95 (92.2)
Lobular	3 (2.9)
Others (metaplastic, papillary, medullary)	5 (4.9)
Histo grade, N (%)	
I	1 (1)
II	33 (32)
III	56 (54.4)
N/A	13 (12.6)
Ki-67, N (%)	
< 20%	10 (9.7)
≥ 20%	89 (86.4)
N/A	4 (3.9)
Site of metastasis, N (%)	
Bone	51 (49.5)
Lung	58 (56.3)
Liver	30 (29.1)
Brain	11 (10.7)
Others (adrenal, soft tissue)	2 (1.9)

*SD* standard deviation, *BMI* body mass index, *NST* no special type

**Fig. 1:**
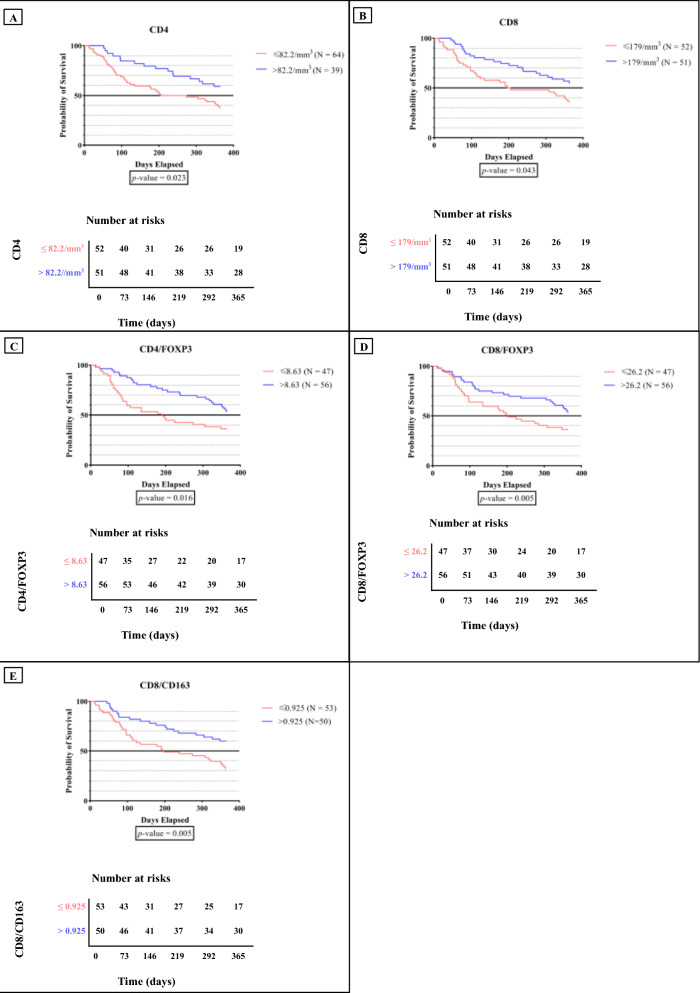
Impact of biomarker expression on 1 year OS of mTNBC patients **A** 1 year OS based on CD4 level. **B** 1 year OS based on CD8 level. **C** 1 year OS based on CD4/FOXP3 level. **D** 1 year OS based on CD8/ FOXP3 level. **E** 1 year OS based on CD8/ CD163 level. Survival curves were analyzed using Kaplan-Meier method and compared using log-rank test. *OS* overall survival, *mTNBC* metastatic triple-negative breast cancer.

Multivariate analysis demonstrated that high CD4/FOXP3 (HR 1.857; 95% CI 1.049–3.288; *p* = 0.034) and CD8/CD163 ratios (HR 2.089; 95% CI 1.174–3.717; *p* = 0.012) significantly improved 1-year OS. In Kaplan–Meier and univariate Cox regression analyses, high levels of CD4, CD8, CD4/FOXP3, CD8/CD163, and CD8/FOXP3 were significantly associated with higher rates of 1 year OS (Table 2, Fig. 1).

**Table 2:** The ROC curve and cox regression analysis of the prognostic markers

Variables	ROC Curve				Univariate cox regression		Multivariate cox regression	
	p-value	Cut-off	Sensitivity (in %)	Specificity (in %)	HR (95% CI)	p-value	HR (95% CI)	p-value
CD4	0.067	82.2/mm^3^	71.4	48.9	1.935 (1.083–3.457)	**0.026**	1.415 (0.638–3.141)	0.393
CD8	0.028	179/mm^3^	58.9	61.7	1.867 (1.095–3.182)	**0.022**	1.621 (0.870–3.021)	0.128
CD4/FOXP3 ratio	0.023	8.63	60.0	61.4	2.020 (1.145–3.564)	**0.015**	1.857 (1.049–3.288)	**0.034**
CD8/FOXP3 ratio	0.038	26.2	60.0	61.4	1.821 (1.032–3.231)	**0.039**	1.467 (0.716–3.006)	0.295
CD8/CD163 ratio	0.017	0.925	64.3	63.8	2.169 (1.254–3.752)	**0.006**	2.089 (1.174–3.717)	**0.012**

Bold indicates a statistically significant

*HR* hazard ratio, *CI* confidence interval

**Fig. 2:**
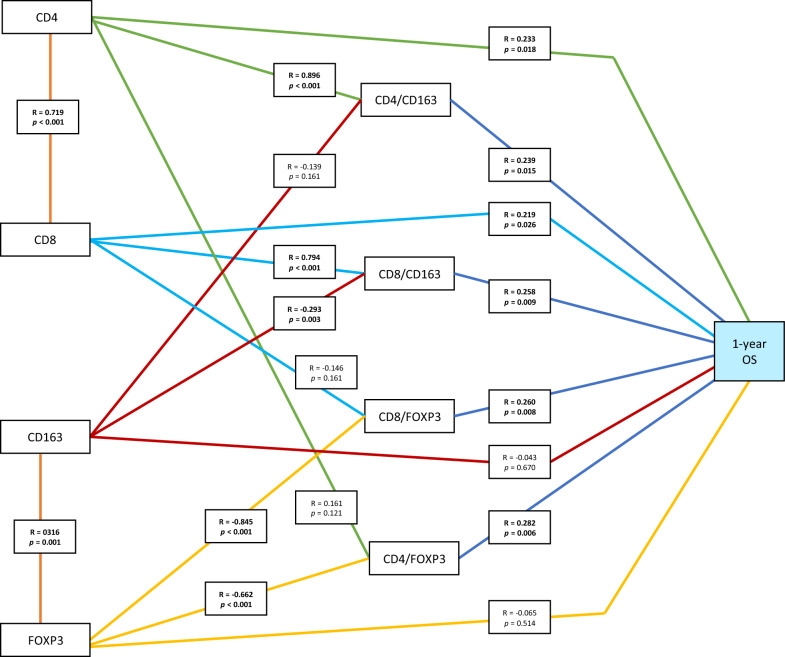
The correlation path analysis between each antitumor and protumor marker towards 1 year OS. *OS* overall survival. Analyzed using Spearman’s correlation test. Bold indicates a statistically significant correlation. Green lines indicate the path of CD4. Light blue lines indicate the path of CD8. Red lines indicate the path of CD163. Yellow lines indicate the path of FOXP3. Orange lines indicate the correlation either between each antitumor or protumor. Dark blue lines indicate the correlation between antitumor/protumor ratio and 1 year OS

In Fig. 2, our path analysis showed that the recursive patterns of antitumor CD4 (R = 0.233, *p* = 0.018) and CD8 cells (R = 0.219, *p* = 0.026) were directly correlated with 1 year OS. Remarkably, 1 year OS had indirect correlations with CD163 and FOXP3 through the antitumor/protumor ratios, including CD4/FOXP3 (R = 0.282; *p* = 0.006), CD4/CD163 (R = 0.239; *p* = 0.015), CD8/FOXP3 (R = 0.260; *p* = 0.008), and CD8/CD163 (R = 0.258; *p* = 0.009). Furthermore, there were significant positive correlations between CD4 and CD4/CD163 (R = 0.896, *p* < 0.001, between CD8 and CD8/CD163 (R = 0.794, *p* < 0.001). There were also significant negative correlations between FOXP3 and CD4/FOXP3 (R = − 0.662, *p* < 0.001); FOXP3 and CD8/FOXP3 (R = − 0.845, *p* < 0.001); and CD163 and CD8/CD163 (R = − 0.293, *p* = 0.03).

## Discussion

To the best of our knowledge, this is the first study that evaluated TME of TNBC in metastatic settings. Based on our multivariate analysis, high CD4/FOXP3 and CD8/CD163 ratios were significantly associated with higher 1 year OS. Kaplan–Meier and univariate Cox regression analyses demonstrated that higher rates of 1 year OS were related with higher levels of CD4, CD8, CD4/FOXP3, CD8/CD163, and CD8/FOXP3 (Table 2 and Fig. 1).

High level of CD4/FOXP3 ratio was significantly associated with a higher rate of 1 year OS (Table 2 and Fig. 1C). The effect of this antitumor/protumor ratio was more robust than the single effect of CD4 (Table 2). A cohort study by Tavares et al. revealed that non-metastatic TNBC patients with low CD4/FOXP3 ratios had significantly reduced OS compared to patients with high ratios [15]. These findings, including ours, reflected the complex interaction between CD4 and FOXP3 in metastatic and non-metastatic TNBC.

According to the current evidence, CD4 T-cells are vital parts of the tumor immunity. They facilitate antitumor response of CD8 T-cells by supporting the proinflammatory cross-presenting dendritic cells (DC) [22–24]. This ultimately provides activating signals for CD8 T-cells, including the development of cytotoxicity and production of tumoricidal cytokines such as interferon-γ (IFN-γ) and tumor necrosis factor-α (TNF-α) [24–27]. On the other hand, FOXP3 has been considered to reduce or prevent inflammation-mediated tumor progression. It may diminish the activity of the CD4 T-cells through cell-to-cell interaction [28–30]. According to a cohort study conducted by Liu et al. it was also found that Treg exhibits immunosuppressive effects in non-small cell lung cancer (NSCLC) patients. Treg plays a significant role in promoting immunosuppressive mechanisms within malignant diseases, as they effectively impacts the immune response against different types of cancer cells [31]. This evidence validates the previous theory regarding the protumor FOXP3 suppressing the antitumor immune cells rather than targeting the cancer cell directly [32]. Thus, a higher level of CD4/FOXP3 can reflect a significant improvement in the 1 year OS (Table 2, Fig. 1C).

High CD8/CD163 ratio was also significantly associated with a higher rate of the 1 year OS (Table 2, Fig. 1E). This antitumor/protumor ratio was greater in significance than the single effect of CD8 or CD163 (Table 2). A cohort study of non-metastatic TNBC patients by Ren et al. showed that patients with high CD8 and low CD163 had significantly better 1 year OS [33]. These results indicate complex interactions between CD8 and CD163 in metastatic and non-metastatic TNBC microenvironments.

Based on the previous studies, CD8 T-cells are the key players in antitumor adaptive immunity for immunological surveillance and tolerance [2]. CD8 T-cells clear cancer cells directly by releasing perforin and granzymes and inducing apoptosis by activating the FasL pathway [2, 34, 35]. On the other hand, CD163 M2-macrophages are a subset of naive M0-macrophage cells that induce the apoptosis of CD8 via the PD-L1 expression; prevent the CD8 T-cells from migrating to the tumor site; and facilitate the tumor progression, metastatis, and angiogenesis via the secretion of the TGF-β, MMP-2, IL-10, and IL-13 [36, 37]. CD163 triggers an immunosuppressive microenvironment and inhibits the antitumor immune response within the TME of TNBC [38]. Thus, the CD8/CD163 ratio can potentially impact the decision-making process regarding mTNBC therapies, specifically harnessing the full capability of the immune system in combating cancer (Table 2, Fig. 1E) [23, 26].

Surprisingly, PD-L1 IHC and PD-L1 mRNA showed no significant effects on the 1-year OS. PD-L1 is an immune checkpoint inhibitor that could potentially reduce antitumor immune cells by binding to PD-1 [36, 39]. The interaction of PD-1/PD-L1 molecules leads to the apoptosis of CD8 T-cells, increases the conversion of T-reg, and protects the macrophages from the destruction by CD8 T-cells [39–41]. Interestingly, another cohort study by Purwanto et al. revealed that high PD-L1 mRNA level significantly worsened the prognosis of non-metastatic TNBC patients [42]. On the other hand, Tavares et al. showed that PD-L1 level had no significance for the prognosis of non-metastatic TNBC [15]. The differences might be accounted due to the complex immune interactions in a metastatic setting.

In the path analysis (Fig. 2**)**, the recursive pattern of antitumor CD4 and CD8 cells was directly correlated with 1 year OS. Interestingly, 1 year OS had indirect correlations with CD163 and FOXP3 through the antitumor/protumor ratios, including CD4/FOXP3, CD4/CD163, CD8/FOXP3, and CD8/CD163. These findings validated the previous theory regarding the mechanism of action of protumor CD163 and FOXP3 which worked indirectly against tumor cells by suppressing the activity of effector cells [32]. Furthermore, the significant positive correlation between CD4 and CD4/CD163 ratio and between CD8 and CD8/CD163 ratio specifically indicated that an increase in the levels of either CD4 or CD8 was associated with an increase in the 1 year OS. The significant negative correlations between FOXP3 and CD4/FOXP3; FOXP3 and CD8/FOXP3; and CD163 and CD8/CD163 also indicated that a decline in the levels of either CD163 or FOXP3 was associated with an increase in 1 year OS. Thus, we concluded that antitumor activity had a stronger impact than protumor activity on the 1 year OS.

**Fig. 3:**
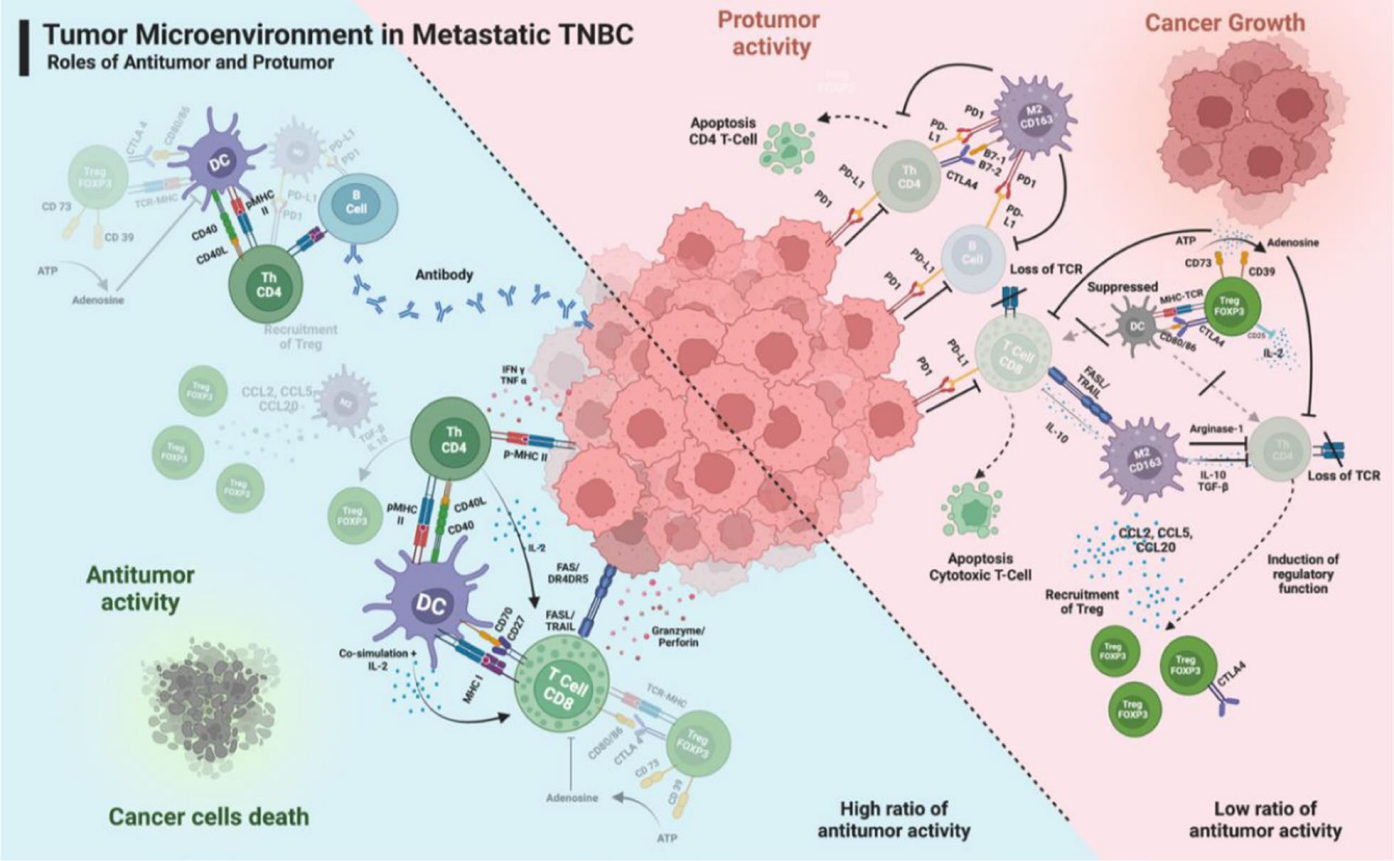
The proposed mechanisms of tumor microenvironment in metastatic TNBC. *DC*,dendritic cells, *MHC* major histocompatibility complex, *TGF-β* transforming growth factor-beta, *Treg* T-regulatory cells, *M2* macrophage type 2, *Th* T-helper cell, *TCR* T-cell receptor TRAIL, tumor necrosis factor-related apoptosis-inducing ligand. The antitumor immune system works directly against cancer cells, whereas the protumor immune system works indirectly by suppressing the antitumor immune system. The activities of CD4 and CD8 immune cells have a direct impact on tumor cells. The activation of DC is initiated by CD4, which subsequently triggers the activation of CD8. CD4 cells induce apoptosis in cancer cells by secreting IFNγ, TNFα, and via p-MHC II, whereas CD8 cells induce apoptosis by producing granzyme, perforin, and activating the FasL/TRAIL pathway. Protumor immune cells of CD163 and FOXP3 exert their effects indirectly by inhibiting the antitumor immune cells mediated by the PD-L1/PD-1, CTLA/B7, FasL/TRAIL pathway, and IL-10 secretion. The CD163 secretes CCL2, CCL5, and CCL20 which attract the FOXP3 cells in the TME. It also secretes IL-10 and TGF-β which suppress the TCR expression. Adenosine induces the apoptosis of CD8 and suppresses the TCR expression. Created with biorender.com. Figure courtesy of Jeffry Beta Tenggara. Permission to reuse the figure in any form must be obtained directly from Jeffry Beta Tenggara.

In accordance with these findings, we propose a mechanism to explain how the antitumor and protumor immune systems work. Antitumor immune system works directly against the cancer cells, whereas protumor immune system works indirectly by inhibiting the antitumor immune system (Fig. 3).

There are several strengths of this study. First, this is the first study to provide the evidence that de novo mTNBC patients who had high levels of CD4/FOXP3 or CD8/CD163 ratios had significantly improved 1-year OS. Second, this is the first study that used the double-stain IHC technique to assess CD4 and FOXP3 in mTNBC to differentiate T-helper cells from the Treg cells. IHC staining has the advantage of being highly accessible and has a greater possibility of clinical applications [43]. Third, we successfully recruited 103 de novo mTNBC patients from different hospitals in Indonesia. In fact, the identification of mTNBC in breast cancer patients is challenging due to its rarity. Consequently, the collection of the samples necessitates an extensive screening [1, 3, 4]. We believe these substantial samples accurately represent the mTNBC population.

The implications of this study can influence not only the TME as a suggestive prognostic marker but also potentially assist healthcare professionals in making personalized and precise treatment decisions.

## Conclusions

This is the first study to demonstrate that high levels of CD4/FOXP3 and CD8/CD163 were significantly associated with the 1 year OS in de novo mTNBC patients. The 1 year OS was directly correlated with CD4 and CD8 and was indirectly correlated with CD163 and FOXP3. Thus, we strongly suggest the introduction of these prognostic markers into clinical practice as their application might be beneficial to maximize treatment in mTNBC.

## Limitations

The potential weakness of the study was in its application which was limited on de novo mTNBC patients. Further research are required for non-de novo mTNBC patients. On the other hand, this multicenter retrospective study has limitations due to differences in local policies across hospitals regarding the determination of metastasis. These different policies are influenced by the capabilities and limitations of each hospital’s facilities. Therefore, it became unreachable to conduct a comprehensive mapping of metastasis data across all samples [44]. Despite this limitation, we can conclusively state that high levels of CD4/FOXP3 and CD8/CD163 significantly improved the 1 year OS in de novo mTNBC patients. Thus, this should be considered to improve the study design in further studies.

### Supplementary Information

**Additional file 1: Fig. S1.** Flowchart of subjects included in the present study.

**Additional file 2: Fig. S2.** IHC staining.

## Data Availability

The dataset analysed during the current study is available in the Figshare repository, https://doi.org/10.6084/m9.figshare.23694270. The unprocessed data are available from the corresponding author on reasonable request.

## Abbreviations

ASCO: American society of clinical oncology
BCR: B-cell receptor
CPS: Combined positive score
DC: Dendritic cells
ER: Estrogen receptors
FasL: Fas ligand
Il: Interleukin
Th: T-helper cell
TNBC: Triple-negative breast cancer
FFPE: Formalin-fixed paraffin-embedded
FISH: Fluorescence in situ hybridization
FOXP3: Forkhead-box-P3
HER2: Human epidermal growth factor receptor 2
IHC: Immunohistochemistry
MHC: Major histocompatibility complex
mRNA: Messenger ribonucleic acid
mTNBC: Metastatic triple-negative breast cancer
NK: Natural killer
OS: Overall survival
*PD-1*: Programmed death-1
*PD-L1*: Programmed death-ligand 1
PR: Progesterone receptor
ROC curve: Receiver operating characteristic curve
*RT-PCR*: Real-time polymerase chain reaction
TCR: T-cell receptor
TGF-β: Transforming growth factor-beta
*TILs*: Tumor-infiltrating lymphocytes
TRAIL: Tumor necrosis factor-related apoptosis-inducing ligand
Treg: Regulatory T-cells
TME: Tumor microenvironment

## References

[1] Almansour NM (2022). Triple-negative breast cancer: a brief review about epidemiology, risk factors, signaling pathways, treatment and role of artificial intelligence. Front Mol Biosci.

[2] Oshi M, Newman S, Tokumaru Y, Yan L, Matsuyama R, Endo I (2020). Inflammation is associated with worse outcome in the whole cohort but with better outcome in triple-negative subtype of breast cancer patients. J Immunol Res.

[3] Zheng H, Siddharth S, Parida S, Wu X, Sharma D (2021). Tumor microenvironment: key players in triple negative breast cancer immunomodulation. Cancers.

[4] Anders CK, Carey LA (2009). Biology, metastatic patterns, and treatment of patients with triple-negative breast cancer. Clin Breast Cancer.

[5] Bou Zerdan M, Ghorayeb T, Saliba F, Allam S, Bou Zerdan M, Yaghi M (2022). Triple negative breast cancer: updates on classification and treatment in 2021. Cancers.

[6] Kassam F, Enright K, Dent R, Dranitsaris G, Myers J, Flynn C (2009). Survival outcomes for patients with metastatic triple-negative breast cancer: implications for clinical practice and trial design. Clin Breast Cancer.

[7] Kazmi S, Chatterjee D, Raju D, Hauser R, Kaufman PA (2020). Overall survival analysis in patients with metastatic breast cancer and liver or lung metastases treated with eribulin, gemcitabine, or capecitabine. Breast Cancer Res Treat.

[8] Benchama O, Malamas MS, Praveen K, Ethier EC, Williams MK, Makriyannis A (2022). Inhibition of triple negative breast cancer-associated inflammation and progression by N- acylethanolamine acid amide hydrolase (NAAA). Sci Rep.

[9] Lan T, Chen L, Wei X (2021). inflammatory cytokines in cancer: comprehensive understanding and clinical progress in gene therapy. Cells.

[10] Salem ML, Attia ZI, Galal SM (2016). Acute inflammation induces immunomodulatory effects on myeloid cells associated with anti-tumor responses in a tumor mouse model. J Adv Res.

[11] Marusyk A, Tabassum DP, Altrock PM, Almendro V, Michor F, Polyak K (2014). Non-cell-autonomous driving of tumour growth supports sub-clonal heterogeneity. Nature.

[12] Lee JM, Lee M-H, Garon E, Goldman JW, Salehi-Rad R, Baratelli FE (2017). Phase I trial of intratumoral injection of *CCL21* gene-modified dendritic cells in lung cancer elicits tumor-specific immune responses and CD8+ T-cell Infiltration. Clin Cancer Res.

[13] Hammond MEH, Hayes DF, Dowsett M, Allred DC, Hagerty KL, Badve S (2010). American society of clinical oncology/college of american pathologists guideline recommendations for immunohistochemical testing of estrogen and progesterone receptors in breast cancer. J Clin Oncol.

[14] Wolff AC, Hammond MEH, Hicks DG, Dowsett M, McShane LM, Allison KH (2013). Recommendations for Human epidermal growth factor receptor 2 testing in breast cancer: American society of clinical oncology/college of American pathologists clinical practice guideline update. J Clin Oncol.

[15] Tavares MC, Sampaio CD, Lima GE, Andrade VP, Gonçalves DG, Macedo MP (2021). A high CD8 to FOXP3 ratio in the tumor stroma and expression of PTEN in tumor cells are associated with improved survival in non-metastatic triple-negative breast carcinoma. BMC Cancer.

[16] Salgado R, Denkert C, Demaria S, Sirtaine N, Klauschen F, Pruneri G (2015). The evaluation of tumor-infiltrating lymphocytes (TILs) in breast cancer: recommendations by an International TILs working group 2014. Ann Oncol.

[17] Yip WK, Abdullah MA, Yusoff SM, Seow HF (2009). Increase in tumour-infiltrating lymphocytes with regulatory T cell immunophenotypes and reduced ζ-chain expression in nasopharyngeal carcinoma patients. Clin Exp Immunol.

[18] Bankhead P, Loughrey MB, Fernández JA, Dombrowski Y, McArt DG, Dunne PD (2017). QuPath: open source software for digital pathology image analysis. Sci Rep.

[19] Park Y, Koh J, Na HY, Kwak Y, Lee K-W, Ahn S-H (2020). PD-L1 testing in gastric cancer by the combined positive score of the 22C3 PharmDx and SP263 assay with clinically relevant cut-offs. Cancer Res Treat.

[20] Yarso K, Bellynda M, Azmiardi A, Wasita B, Heriyanto D, Astuti I (2021). Chemotherapy negates the effect of SDF1 mRNA to distant metastasis and poor overall survival in breast cancer patients. Asian Pac J Cancer Prev.

[21] Lechner MG, Liebertz DJ, Epstein AL (2010). Characterization of cytokine-induced myeloid-derived suppressor cells from normal human peripheral blood mononuclear cells. J Immunol.

[22] National Comprehensive Cancer Network. NCCN Guidelines. 2022. https://www.nccn.org/guidelines/category_1. Accessed 19 Nov 2022.

[23] Tay RE, Richardson EK, Toh HC (2021). Revisiting the role of CD4+ T cells in cancer immunotherapy—new insights into old paradigms. Cancer Gene Ther.

[24] Borst J, Ahrends T, Bąbała N, Melief CJM, Kastenmüller W (2018). CD4+ T cell help in cancer immunology and immunotherapy. Nat Rev Immunol.

[25] Hor JL, Whitney PG, Zaid A, Brooks AG, Heath WR, Mueller SN (2015). Spatiotemporally distinct interactions with dendritic cell subsets facilitates CD4+ and CD8+ T cell activation to localized viral infection. Immunity.

[26] Smith CM, Wilson NS, Waithman J, Villadangos JA, Carbone FR, Heath WR (2004). Cognate CD4+ T cell licensing of dendritic cells in CD8+ T cell immunity. Nat Immunol.

[27] Laidlaw BJ, Craft JE, Kaech SM (2016). The multifaceted role of CD4+ T cells in CD8+ T cell memory. Nat Rev Immunol.

[28] Kennedy R, Celis E (2008). Multiple roles for CD4 ^+^ T cells in anti-tumor immune responses. Immunol Rev.

[29] Jackute J, Zemaitis M, Pranys D, Sitkauskiene B, Miliauskas S, Bajoriunas V (2015). The prognostic influence of tumor infiltrating Foxp3+CD4+, CD4+ and CD8+ T cells in resected non-small cell lung cancer. J Inflamm.

[30] Mandapathil M, Szczepanski MJ, Szajnik M, Ren J, Lenzner DE, Jackson EK (2009). increased ectonucleotidase expression and activity in regulatory T cells of patients with head and neck cancer. Clin Cancer Res.

[31] Liu C, Sun B, Hu X, Zhang Y, Wang Q, Yue J (2019). Stereotactic ablative radiation therapy for pulmonary recurrence-based oligometastatic non-small cell lung cancer: survival and prognostic value of regulatory T cells. Int J Radiation Oncol Biol Phys.

[32] Togashi Y, Shitara K, Nishikawa H (2019). Regulatory T cells in cancer immunosuppression — implications for anticancer therapy. Nat Rev Clin Oncol.

[33] Ren X, Song Y, Pang J, Chen L, Zhou L, Liang Z (2023). Prognostic value of various immune cells and Immunoscore in triple-negative breast cancer. Front Immunol.

[34] Farhood B, Najafi M, Mortezaee K (2019). CD8 ^+^ cytotoxic T lymphocytes in cancer immunotherapy: a review. J Cell Physiol.

[35] Zhang L, Zhang W, Li Z, Lin S, Zheng T, Hao B (2022). Mitochondria dysfunction in CD8+ T cells as an important contributing factor for cancer development and a potential target for cancer treatment: a review. J Exp Clin Cancer Res.

[36] Hudson K, Cross N, Jordan-Mahy N, Leyland R (2020). The extrinsic and intrinsic roles of PD-L1 and Its receptor PD-1: implications for immunotherapy treatment. Front Immunol..

[37] Thorsson V, Gibbs DL, Brown SD, Wolf D, Bortone DS, Ou Yang T-H (2018). The immune landscape of cancer. Immunity.

[38] Zhu Y, Zhang H, Pan C, He G, Cui X, Yu X (2023). Integrated tumor genomic and immune microenvironment analysis identifies predictive biomarkers associated with the efficacy of neoadjuvant therapy for triple-negative breast cancer. Cancer Med.

[39] Jiang X, Wang J, Deng X, Xiong F, Ge J, Xiang B (2019). Role of the tumor microenvironment in PD-L1/PD-1-mediated tumor immune escape. Mol Cancer.

[40] Medrek C, Pontén F, Jirström K, Leandersson K (2012). The presence of tumor associated macrophages in tumor stroma as a prognostic marker for breast cancer patients. BMC Cancer.

[41] Ohaegbulam KC, Assal A, Lazar-Molnar E, Yao Y, Zang X (2015). Human cancer immunotherapy with antibodies to the PD-1 and PD-L1 pathway. Trends Mol Med.

[42] Purwanto I, Heriyanto DS, Ghozali A, Widodo I, Dwiprahasto I, Aryandono T (2020). Overexpression of programmed death-ligand 1 receptor MRNA as an independent negative prognostic factor for triple negative breast cancer. World J Oncol.

[43] Magaki S, Hojat SA, Wei B, So A, Yong WH (2019). An introduction to the performance of immunohistochemistry.

[44] Wang R, Zhu Y, Liu X, Liao X, He J, Niu L (2019). The clinicopathological features and survival outcomes of patients with different metastatic sites in stage IV breast cancer. BMC Cancer.

