# Supplementary material for: The relationship between high ratios of CD4/FOXP3 and CD8/CD163 and the improved survivability of metastatic triple-negative breast cancer patients: a multicenter cohort study
Source: BMC Res Notes. 2024 Feb 2;17:44. doi: 10.1186/s13104-024-06704-z (PMC10835864; doi:10.1186/s13104-024-06704-z)
Supplement: Supplementary file 1 — Additional file 1: Fig. S1. Flowchart of subjects included in the present study. [file 13104_2024_6704_MOESM1_ESM.pdf]

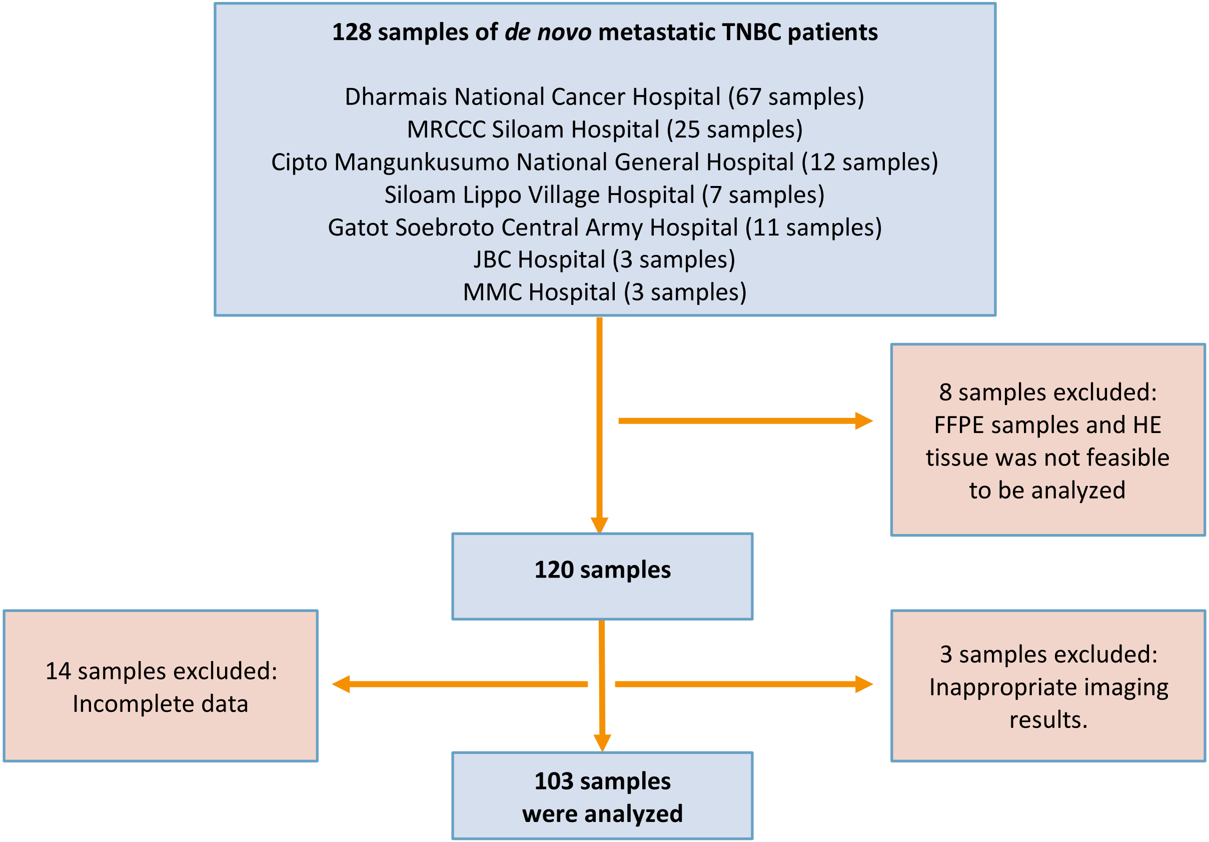

Additional file 1 : Fig. S1. Flowchart of subjects included in the present study

Abbreviations: FFPE, formalin-fixed paraffin-embedded; HE, hematoxylin and eosin
