# Supplementary material for: The relationship between high ratios of CD4/FOXP3 and CD8/CD163 and the improved survivability of metastatic triple-negative breast cancer patients: a multicenter cohort study
Source: BMC Res Notes. 2024 Feb 2;17:44. doi: 10.1186/s13104-024-06704-z (PMC10835864; doi:10.1186/s13104-024-06704-z)
Supplement: Supplementary file 2 — Additional file 2: Fig. S2. IHC staining. [file 13104_2024_6704_MOESM2_ESM.pdf]

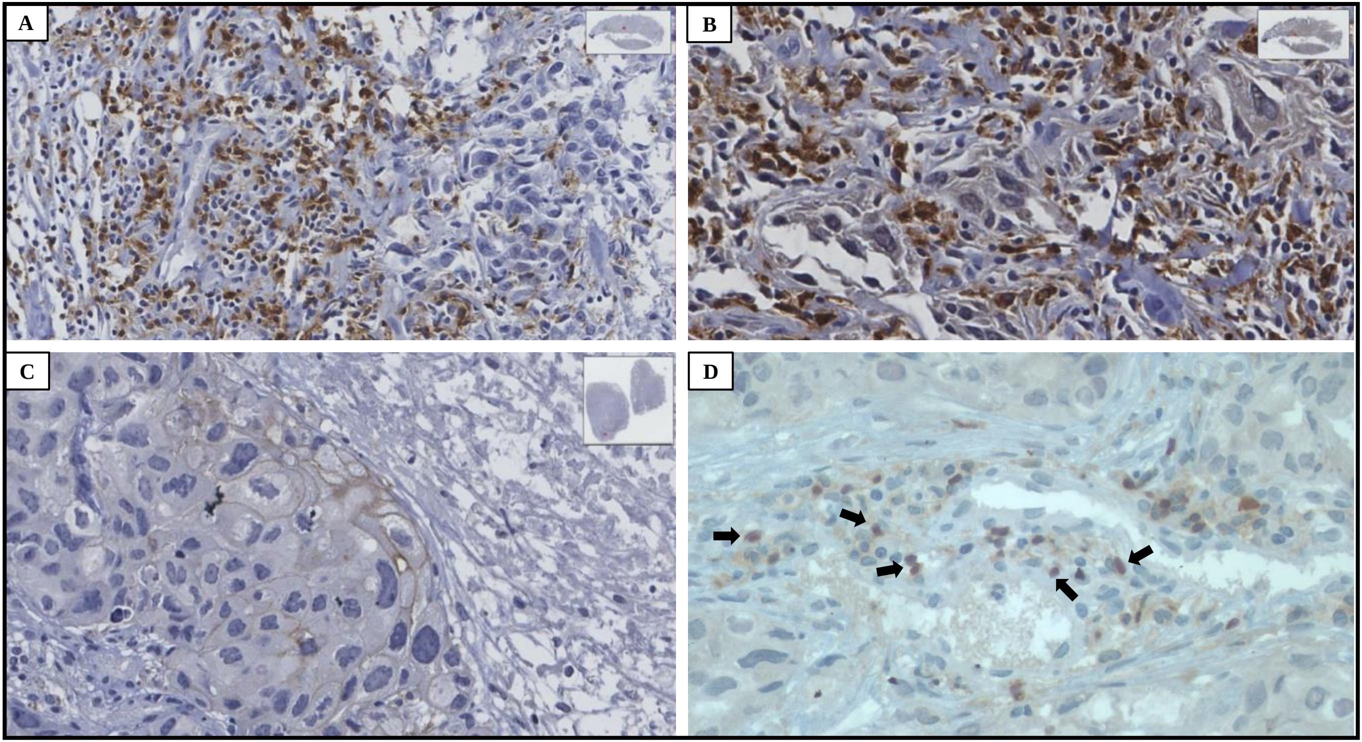

Additional file 2 : Fig. S2. IHC staining.

2A) IHC staining of CD8 (200x). 2B) IHC Staining of CD163 (200x). 2C) IHC Staining of PDL-1 (200x). 2D) IHC Double staining of CD4 and FOXP3 (200x); Arrows indicate FOXP3 cell nuclei (red).

Abbreviations: IHC, immunohistochemistry; FOXP3, forkhead box P3.
